# Improved Dissolution Rate and Intestinal Absorption of Fexofenadine Hydrochloride by the Preparation of Solid Dispersions: In Vitro and In Situ Evaluation

**DOI:** 10.3390/pharmaceutics13030310

**Published:** 2021-02-27

**Authors:** Basanth Babu Eedara, Dinesh Nyavanandi, Sagar Narala, Prabhakar Reddy Veerareddy, Suresh Bandari

**Affiliations:** Department of Pharmaceutics, St. Peter’s Institute of Pharmaceutical Sciences, Vidyanagar, Warangal 506001, India; dnyavana@go.olemiss.edu (D.N.); snarala@go.olemiss.edu (S.N.); vpreddyidia@gmail.com (P.R.V.); sbandari@olemiss.edu (S.B.)

**Keywords:** fexofenadine hydrochloride, PEG 20,000, poloxamer188, dissolution, absorption

## Abstract

The objective of this study was to enhance dissolution and permeation of a low soluble, absorbable fexofenadine hydrochloride (FFH) by preparing solid dispersions using polyethylene glycol 20,000 (PEG 20,000) and poloxamer 188 as carriers. The phase solubility measurement for the supplied FFH revealed a linear increase in the solubility of fexofenadine with increasing carrier concentration in water (1.45 mg/mL to 11.78 mg/mL with 0% *w/v* to 30% *w/v* PEG 20,000; 1.45 mg/mL to 12.27 mg/mL with 0% *w/v* to 30% *w/v* poloxamer 188). To select the appropriate drug carrier concentration, a series of solid dispersions were prepared in the drug carrier weight ratios of 1:1, 1:2 and 1:4 by fusion method. The solid dispersions composed of drug carrier at 1:4 weight ratio showed highest dissolution with the time required for the release of 50% of the drug <15 min compared to the supplied FFH (>120 min). The intestinal absorption study presented a significant improvement in the absorption of drug from the solid dispersions composed of poloxamer 188 than PEG 20,000. In summary, the solid dispersions of FFH prepared using PEG 20,000 and poloxamer 188 demonstrated improved dissolution and absorption than supplied FFH and could be used to improve the oral bioavailability of fexofenadine.

## 1. Introduction

Allergic rhinitis (AR), an immunoglobulin E-mediated inflammation of the nasal mucosa, is estimated to affect approximately 10% to 25% of the worldwide population [1]. The pathophysiology of AR involves the release of several proinflammatory mediators. Histamine is one of the key proinflammatory mediators released by the mast cells, which stimulates H1- receptors and leads to the characteristic symptoms of rhinorrhea, nasal itching, sneezing, and nasal congestion [2]. Oral antihistamines have been the first-line treatment for AR over decades and are effective at treating the symptoms caused by allergen exposure [2].

Fexofenadine hydrochloride (FFH) is a safe, long acting, non-sedating anti-histamine with selective peripheral H1- receptor antagonist activity used in allergic conditions like seasonal allergic rhinitis and chronic idiopathic urticaria [3]. It is a white to off-white crystalline powder and its oral bioavailability in humans is about 33% [4]. The low oral bioavailability of fexofenadine is attributed to the slightly soluble nature in water, low intestinal permeability, and active efflux by intestinal P-glycoprotein (P-gp) [5,6].

Several formulation strategies such as crystal modification (salts, polymorphs, and co-crystals), surfactant systems, particle size reduction, amorphization, cyclodextrin complexation, lipid formulations and pH modification have been followed to enhance the oral bioavailability of poorly absorbed drugs [7,8,9,10,11,12,13,14,15,16]. Among these formulations, the solid dispersions are easy to prepare, and extensively used approach to enhance the solubility and dissolution behavior of low soluble drugs as it can produce a solid dosage formulation of a drug in an amorphous or mono-molecularly dispersed state surrounded by an inert carrier [7]. Moreover, solid dispersion approach can also be used to enhance the intestinal permeability of poorly absorbable drugs by using a surface active or lipid carrier [17].

In the literature, several formulation approaches such as disintegrating tablets [18], water-in-oil micro emulsions [19], and phospholipid complexation [20] have been reported to improve the oral bioavailability of fexofenadine. In our previous study, Gelucire 44/14 and d-α-tocopheryl polyethylene glycol 1000 succinate (TPGS) have been used as carriers for the preparation of dispersions and the results showed a significant improvement in the dissolution as well as intestinal permeability of FFH [21]. However, the developed dispersions were sticky due to the semisolid nature of the carriers at room temperature. The current study aimed to develop non-sticky solid dispersions of FFH using polyethylene glycol 20,000 (PEG 20,000) and poloxamer 188 as carriers.

Polyethylene glycol (PEG) is a water-soluble, biocompatible, nonpolar polymer of ethylene oxide widely used in a variety of pharmaceutical formulations. They are available over a wide range of molecular weights from 200 Da to 35,000 Da. High molecular weight PEGs shows greater amphiphilic character [22]. Previous reports demonstrated the P-gp inhibitory activity of PEG and increased the absorption of P-gp substrate drugs [23,24]. Shen et al. demonstrated that the intestinal absorption of quinidine was significantly improved in the presence of PEG 20,000 by inhibiting P-gp activity [25].

Poloxamers are nonionic amphiphilic triblock copolymers composed of a central hydrophobic poly(propylene oxide) (PPO) chain linked with two hydrophilic poly(ethylene oxide) (PEO) chains [26]. Poloxamers have been used as a surfactant, solubilizing agent, wetting agent, and stabilizing agent in various pharmaceutical formulations [27]. Several studies reported the use of poloxamer 188 (molecular weight 8400 Da) as a carrier for the preparation of solid dispersions and has been improved the solubility and dissolution of low water-soluble drugs [28,29]. Furthermore, it is known to inhibit P-gp-mediated efflux activity and enhance the permeability of several P-gp substrate drugs across the intestine [30].

The low melting points of PEG 20,000 (60 to 63 °C) and poloxamer 188 (~50 °C) are also beneficial in the preparation of solid dispersions by the fusion method. The solid dispersions were produced by varying the FFH to carrier (PEG 20,000/poloxamer 188) weight ratios (1:1, 1:2 and 1:4) following the fusion method. Furthermore, this study investigated the influence of PEG 20,000 and poloxamer 188 on the solid-state nature of FFH and their ability to improve the drug solubility, dissolution behavior in water and intestinal absorption rate in rats.

## 2. Materials and Methods

### 2.1. Materials

Fexofenadine hydrochloride (FFH) was obtained as a generous gift sample from Ami Lifesciences Pvt. Ltd. (Vadodara, India). Polyethylene glycol 20,000 (PEG 20,000) was purchased from E. Merck (India) Ltd. (Mumbai, India). Poloxamer 188 was donated by BASF SE (Ludwigshafen, Germany). All other reagents and chemicals used were of analytical or high-performance liquid chromatography (HPLC) grade.

### 2.2. Animals

Healthy male Wistar rats (age: 10 weeks, body weight: 250–300 g) were purchased from Mahaveera Enterprises (146-CPCSEA no:199; Hyderabad, India), acclimatized for at least one week in separate cages in a clean room and maintained under the controlled conditions of temperature with free access to food and water. All the experimental protocols including animal care and handling were reviewed and approved (1003/SPIPS/Wgl/IAEC/2011; 30 April 2011) by the Institutional Animal Ethical Committee, St. Peter’s Institute of Pharmaceutical Sciences (Hanamkonda, India).

### 2.3. Drug Quantification by HPLC

FFH was quantified using a validated HPLC method [21]. Briefly, a Shimadzu (Shimadzu Corporation, Kyoto, Japan) HPLC system equipped with LC-10 AT solvent delivery unit, SPD-10 AVP UV-spectrophotometric detector with Lichrospher C18 column (5 µm, 4.6 × 250 mm was used. The system was operated under isocratic flow at 1 mL/min using a mobile phase consisting of a mixture of 0.05M potassium dihydrogen orthophosphate buffer containing 0.5% *v/v* triethylamine (pH 4.2 adjusted with orthophosphoric acid), acetonitrile, and methanol (50:38:12 *v/v/v*). Samples of 20 µL were injected using an auto sampler and analyzed at a wavelength of 220 nm. The calibration curve was constructed over the concentration range of 0.25–16 µg/mL and was linear with a good correlation coefficient (R^2^ > 0.9996). The limit of detection (LOD) and limit of quantification (LOQ) of FFH were 0.125 µg/mL and 0.25 µg/mL, respectively.

### 2.4. Phase Solubility Study

Solubility of FFH in aqueous solution containing PEG 20,000 and poloxamer 188 was determined according to the method reported by Higuchi and Connors [31]. An excess amount (300 mg) of FFH was mixed with 10 mL of aqueous solutions containing carrier (PEG 20,000 and poloxamer 188) at 5%, 10%, 15%, 20%, 25%, and 30% *w/v* in screw-capped glass vials. These dispersions were maintained at 37 °C in a thermostatically controlled water bath with continuous shaking for 48 h. The contents of each vial were filtered through 0.45 µm membrane filter and the filtrate obtained was analyzed by HPLC after appropriate dilution using methanol.

The apparent stability constant (K_s_), between drug–carrier combination was calculated from the slope of the phase solubility diagrams i.e., drug solubility change with increasing carrier concentration, using the following equation (Equation (1)).

(1) $${\;K}_{s}=\frac{\mathrm{Slope}}{S_{0}}\left(1-\mathrm{Slope}\right)$$

where S_0_ is the solubility of FFH in water, in the absence of carrier.

The Gibbs-free energy of transfer (ΔG°_tr_) of FFH from plain water without carrier to the aqueous solution of carrier was calculated using the following equation (Equation (2)).

(2) $$\Delta G_{\mathrm{tr}}^{{}^{\circ}}=-2.303\mathrm{RT}\;\mathrm{Log}\frac{S_{S}}{S_{0}}$$

where S_S_ and S_0_ are the molar solubility of FFH in an aqueous solution of the carrier and the plain water without carrier, respectively. R is the general gas constant (R = 8.31 J K^−1^ mol^−1^) while T is the temperature in degree Kelvin.

### 2.5. Preparation of Solid Dispersions and Physical Mixtures

Solid dispersions of FFH were prepared using PEG 20,000 and poloxamer 188 as carriers in 1:1, 1:2 and 1:4 weight ratios by fusion method. Briefly, an accurately weighed amount of carrier was melted in a porcelain dish using a water bath maintained at a temperature of 65 °C. The pre-weighed amount of FFH was then added to the molten carrier and mixed thoroughly using a glass stirrer for 5 min to get a uniform dispersion. The resultant mixture was cooled overnight at room temperature. The hardened solid masses were ground in a glass mortar, passes through 250 µm sieve (mesh size #60), transferred into a screw-capped glass vial and stored at room temperature (22 ± 1 °C) in a desiccator with silica gel (30 ± 2% relative humidity).

Physical mixtures of FFH and carriers (PEG 20,000/poloxamer 188) at 1:1, 1:2 and 1:4 weight ratios were gently mixed in a glass mortar with a spatula for 5 min, transferred into a screw-capped glass vial and stored at room temperature (22 ± 1 °C) in a desiccator with silica gel (30 ± 2% relative humidity).

### 2.6. Drug Content Estimation

Drug content in the produced solid dispersions and physical mixtures was estimated by dissolving the powder equivalent to 10 mg of FFH in 100 mL of methanol. An aliquot of sample was centrifuged at 10,000 rpm for 15 min. The supernatant samples were filtered through 0.45 μm membrane filter and the filtrates obtained were analyzed by HPLC after appropriate dilution into the validated range with methanol.

### 2.7. In Vitro Dissolution Studies

The dissolution studies of supplied FFH, solid dispersions and physical mixtures (equivalent to 30 mg of FFH) were conducted using United States Pharmacopeia type II (paddle) dissolution apparatus (Electrolab, Mumbai, India). The dissolution study was conducted using 900 mL of distilled water in a standard, hemispherical bottomed dissolution vessel at the paddle rotation speed of 50 rpm. Aliquots of 5 mL were withdrawn at different time intervals from 15 min to 120 min with fresh medium replacement, immediately filtered using a 0.45 μm filter and quantified by HPLC after appropriate dilution with the mobile phase.

Various parameters such as Q_15_ and Q_120_ i.e., percent drug released at 15 min and 120 min, dissolution efficiency at 15 min and 120 min (DE_15_ and DE_120_), and time taken for 50% release (t_50%_) were calculated as reported in our previous publication [21] to assess the dissolution behavior of solid dispersions in relation to the supplied drug and physical mixture.

### 2.8. Characterization of the Physicochemical Properties

#### 2.8.1. Scanning Electron Microscopy (SEM)

The surface morphologies of supplied FFH, PEG 20,000, poloxamer 188, optimized solid dispersions and their respective physical mixtures were examined using a scanning electron microscope (SEM, Jeol Corporation, Tokyo, Japan) operating at 15 Kv. The powder sample was directly sprinkled onto the double-sided adhesive tape which was affixed to aluminum stub and made electrically conductive by coating them with platinum (approximately 5 nm) in vacuum for 100 s at 30 W. Samples were observed under SEM and micrographs were recorded at different magnifications to study the surface characteristics.

#### 2.8.2. Differential Scanning Calorimetry (DSC)

DSC thermograms of supplied FFH, PEG 20,000, poloxamer 188, optimized solid dispersions, and their respective physical mixtures were obtained using a differential scanning calorimeter unit (TA-60WSI, Shimadzu, Kyoto, Japan) calibrated with sapphire (heat capacity) and indium (heat flow) standards. Powder sample (~4 mg) was weighed in a flat-bottomed aluminum pan (Shimadzu DSC-60, Kyoto, Japan) and press sealed with a standard aluminum lid. All the samples were scanned at a heating rate of 10 °C/min from 30 °C to 300 °C using an empty pan as a reference. Nitrogen gas was purged at a flow rate of 80 mL/min. The heat of fusion of samples was calculated from the peak area of the melting endotherm. 2.8.3. X-ray powder diffraction (XRPD).

X-ray powder diffractograms of supplied FFH, PEG 20,000, poloxamer 188, optimized solid dispersions and their respective physical mixtures were collected using an X’Pert PRO MPD diffractometer (PANalytical, Almelo, The Netherlands) with a copper anode (Cu Kα radiation, λ = 0.15406 nm, 45 kV, 40 mA). Powder samples were gently pressed on an aluminum sample holder and scanned over the range of 3° < 2θ < 50° 2θ at a scanning speed of 5° 2θ/min ambient temperature.

#### 2.8.3. Fourier Transform Infrared Spectroscopy (FTIR)

FTIR spectra of supplied FFH, PEG 20,000, poloxamer 188, optimized solid dispersions and their respective physical mixtures were recorded using FTIR spectrophotometer (Spectrum GX-FT-IR, PerkinElmer Inc., Waltham, MA, USA). Powder samples (~2 mg) were gently mixed with IR grade dry potassium bromide (200 mg) and compressed in a hydraulic press to form disks. The spectrum was recorded as an average of 64 scans in the frequency range of 4000 cm^−1^ to 400 cm^−1^ with the resolution of 4 cm^−1^ at room temperature.

### 2.9. In Situ Intestinal Absorption Study

The in situ intestinal absorption of fexofenadine from the prepared solid dispersions was investigated following the single pass intestinal absorption (SPIP) study as reported previously [32]. Healthy male Wistar rats (age: 10 weeks, body weight: 250 g to 300 g) were fasted overnight before the experiment with free access to water. After anesthesia via intraperitoneal injection of thiopental sodium (50 mg/kg), rats were placed in a supine position on a heating pad to maintain normal body temperature. The abdomen was cut open along the midline and a 10 cm segment of the proximal rat jejunum was selected. Small “V” shape incisions were made at both ends of the selected segment, rinsed with the physiological saline (37 °C), cannulated with perfusion tubing, ligated with a cotton thread, and connected to a syringe pump (Harvard Apparatus PHD 2000 pump, Holliston, MA, USA). The surgical area was kept hydrated by covering with a cotton gauze wetted with physiological saline. Using syringe pump, the intestinal segments were perfused with blank perfusion buffer (PBS, pH 7.4 at 37 °C) for 15 min at 0.5 mL/min flow rate to clear out the intestinal content. The perfusion buffer containing known concentrations (5 mg/mL) of drug (supplied FFH and solid dispersions dispersed in perfusion buffer) was then perfused at a flow rate of 0.2 mL/min for 20 min to reach an initial steady state according to the previous reports [33]. After equilibration, samples were collected at every 15 min for a 90 min perfusion period and frozen at −20 °C until analysis by HPLC.

At the end of the study, animals were euthanized with a cardiac injection of saturated potassium chloride solution. The intestinal segments were excised, and their length and perimeter were measured. Then, the intestinal segments were cut open, washed with blank perfusate buffer, placed in a formaldehyde saline solution (10% *v/v*) for 24 h and embedded in paraffin wax. The paraffin-embedded sections were stained with hematoxylin/eosin and observed under microscope for histological evaluation.

Various permeability parameters such as effective permeability coefficient in rats (P_eff(rat)_, cm/s), predicted effective permeability coefficient in human (P_eff(human)_, cm/s), absorption rate constant (K_a_), and enhancement ratio (ER) were calculated as reported in our previous publication [21] to assess the absorption behavior of fexofenadine from solid dispersions in relation to the supplied drug.

### 2.10. Statistical Analysis

All the experimental data were expressed as mean ± SD (*n* = 3). Statistical analyses were performed by one-way analysis of variance (ANOVA) with Student-Newman-Keuls posthoc testing using GraphPad Prism 5 software (GraphPad Software, San Diego, CA, USA) with *p* ≤ 0.05 as the level of significance.

## 3. Results

### 3.1. Phase Solubility Study

The phase solubility of FFH in water alone and with PEG 20,000/poloxamer 188 (0% to 30% *w/v*) at 37 °C is shown in Table 1. Also, a phase solubility diagram of solubility of FFH (mg/mL) against carrier concentration (% *w/v* of PEG 20,000/poloxamer 188) was plotted (Figure S1, supplementary information) [31]. The solubility of supplied FFH in water at 37 °C was found to be 1.45 ± 0.15 mg/mL. A linear increase in FFH solubility was observed with increasing carrier (PEG 20,000/poloxamer 188) concentration and the solubility curves represented an A_L_ type with the slope less than unity (Figure S1, supplementary information). The solubility of FFH increased by almost seven times in the presence of PEG 20,000/poloxamer 188 in water at a concentration of 30% *w/v* with an R^2^ value > 0.99. This indicated that both the carriers could efficiently increase the solubility of FFH owing to the wettability effect as well as hydrophilic or surface active-like properties [26,28,34,35,36].

Gibbs-free energy is the function of energy formation after the phase transformation of insoluble drug into soluble form. The ΔG°_tr_ value indicates the favorable or unfavorable nature of carrier for drug solubilization in an aqueous medium. The ΔG°_tr_ values (Table 1) of FFH were negative and decreased with increasing carrier concentration which indicated the spontaneous nature of the drug solubilization process in the presence of PEG 20,000 and poloxamer 188 [37]. The values of apparent stability constant (K_s_) calculated using Equation (1) for FFH-PEG 20,000 and FFH- poloxamer 188 combinations were 145.1 mL/g and 118.5 mL/g, respectively which indicated a strong binding affinity between fexofenadine and the carrier [38].

### 3.2. Drug Content

The drug content of all prepared physical mixtures (1:4 *w/w*) and the solid dispersions (1:1 *w/w*, 1:2 *w/w* and 1:4 *w/w*) of FFH with PEG 20,000/poloxamer 188 was found to be 95.2 ± 3% to 99.3 ± 2%. All the solid dispersions showed the presence of high drug content which indicated better suitability of the fusion method used for the preparation of solid dispersions with high content uniformity.

### 3.3. In Vitro Dissolution Study

**Figure 1:**
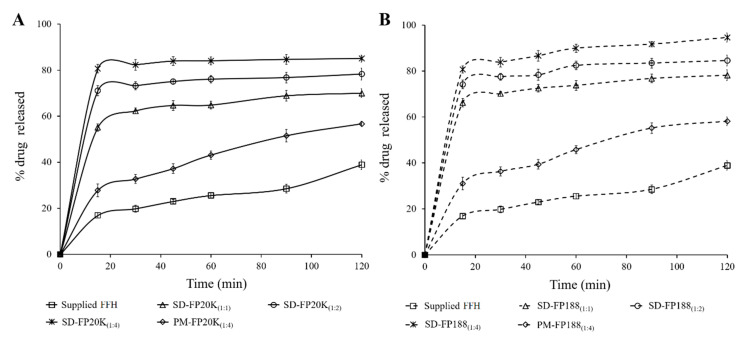
(**A**) In vitro dissolution profiles of supplied FFH, solid dispersions of FFH with PEG 20,000 at 1:1 (SD-FP20K_(1:1)_), 1:2 (SD-FP20K_(1:2)_) and 1:4 (SD-FP20K_(1:4)_) weight ratios, and physical mixture of FFH with PEG 20,000 at 1:4 weight ratio (PM-FP20K_(1:4)_). (**B**) In vitro dissolution profiles of supplied FFH, solid dispersions of FFH with poloxamer 188 at 1:1 (SD-FP188_(1:1)_), 1:2 (SD-FP188_(1:2)_) and 1:4 (SD-FP188_(1:4)_) weight ratios, and physical mixture of FFH with PEG 20,000 at 1:4 weight ratio (PM-FP188_(1:4)_) Each point represents the mean ± SD. (*n* = 3).

Figure 1 shows the dissolution profiles of supplied FFH, the solid dispersions (SD-FP20K_(1:1)_, SD-FP20K_(1:2)_, SD-FP20K_(1:4)_, SD-FP188_(1:1)_, SD-FP188_(1:2)_, and SD-FP188_(1:4)_) and the physical mixtures (PM-FP20K_(1:4)_ and PM-FP188_(1:4)_) prepared using PEG 20,000 and poloxamer 188 in distilled water as dissolution media. The supplied FFH has the lowest dissolution (38.9 ± 2.1%) compared to the solid dispersions and physical mixtures prepared. It was noted that the solid dispersions showed improved dissolution with increasing amounts of PEG 20,000/poloxamer 188 (with PEG 20,000: SD-FP20K_(1:1)_—70.0 ± 1.9% < SD-FP20K_(1:2)_—78.3 ± 2.6% < SD-FP20K_(1:4)_—80.6 ± 1.8%; with poloxamer 188: SD-FP188_(1:1)_—78.2 ± 2.3% < SD-FP188_(1:2)_—84.7 ± 2.3% < SD-FP188_(1:4)_—94.7 ± 1.9%).

Dissolution parameters, represented as the percentage of the drug released (Q_15_ and Q_120_), the percentage dissolution efficiency (DE_15_ and DE_120_), as well as the time required for the release of 50% of the drug (t_50%_) were calculated and presented in Table 2. Supplied FFH showed poor dissolution with Q_15_ of only 16.9%. The overall amount of the drug dissolved from supplied FFH after 120 min was 38.9%, while DE_15_ and DE_120_ were 8.5% and 24.3%, respectively. All the solid dispersions and physical mixtures prepared using PEG 20,000/poloxamer 188 showed significant (*p* < 0.05) differences in FFH dissolution parameters (Table 2).

Generally, the solid dispersions composed of hydrophilic or surface-active carriers improve the solubility and dissolution rate of poorly water-soluble drugs by reduction of the drug particle size, increase in surface area, by changing the crystalline form of the drug to amorphous form, and/or by enhancement of the drug wettability [39]. Poloxamer 188 is an amphiphilic block co-polymer consisting of ethylene oxide and propylene oxide blocks, which can self-aggregates to form micelles in aqueous solution and helps in solubilization of a hydrophobic drug [26]. Polyethylene glycols (PEGs) are polymers of ethylene oxide which improved the solubility and dissolution of FFH in solid dispersion may be due to the formation of a hydrophilic film of PEG around the drug particles which reduces the hydrophobic interaction of drug and improves the wettability [40].

**Figure 2:**
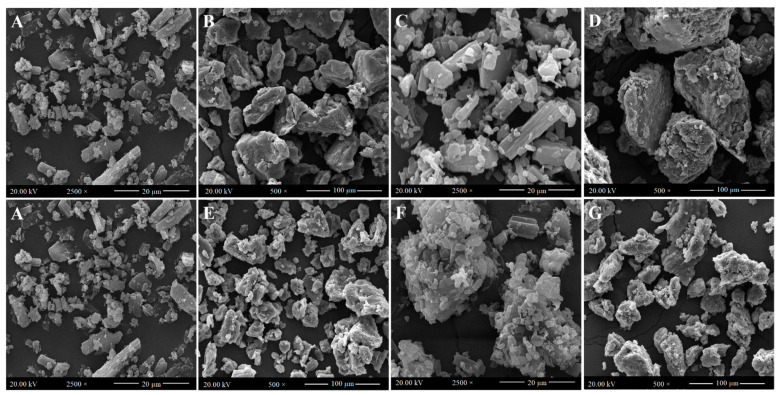
Scanning electron microscopic images of (**A**) Supplied FFH, (**B**) Supplied PEG 20,000, (**C**) Physical mixture of FFH with PEG 20,000 at 1:4 ratio (PM-FP20K_(1:4)_), (**D**) Solid dispersion of FFH with PEG 20,000 at 1:4 ratio (SD-FP20K_(1:4)_), (**E**) Supplied poloxamer 188, (**F**) Physical mixture of FFH with poloxamer 188 at 1:4 ratio (PM-FP188_(1:4)_), (**G**) Solid dispersion of FFH with poloxamer 188 at 1:4 ratio (SD-FP188_(1:4)_).

Similarly, the enhanced drug dissolution obtained for physical mixtures (PM-FP20K_(1:4)_ and PM-FP188_(1:4)_) could be attributed to the wetting effect, and solubilization of poloxamer 188 and PEG 20,000. In the physical mixture, drug particles were intermixed or adhered on to the surface of the carrier particles (Figure 2). During dissolution, the carrier particles might have hydrated rapidly into a carrier solution, due to their hydrophilic/surface active nature, which improves the wetting and solubilization of the adjacent drug particles into the medium [34].

### 3.4. Solid State Characterization

#### 3.4.1. Scanning Electron Microscopy

Figure 2 shows the representative SEM images of supplied FFH, PEG 20,000, poloxamer 188, the physical mixtures (PM-FP20K_(1:4)_ and PM-FP188_(1:4)_) and the solid ispersions (SD-FP20K_(1:4)_ and SD-FP188_(1:4)_). Supplied FFH was appeared as smooth surfaced crystalline particles of irregular size and shape adsorbed with tiny particles. The PEG 20,000 and poloxamer 188 showed large particles of irregular size and shape with blunt edges. Physical mixtures (PM-FP20K_(1:4)_ and PM-FP188_(1:4)_) contained FFH crystalline particles adsorbed on the surface of the respective carrier particles (PEG 20,000/poloxamer 188). The solid dispersions (SD-FP20K_(1:4)_ and SD-FP188_(1:4)_) appeared to be agglomerated particles of irregular size and shape with rough surface, but the crystalline drug particles or separate phases were indistinguishable.

In our previous study, Gelucire 44/14 and TPGS 1000 have been used as carriers in the preparation of the lipid surfactant-based dispersions of FFH and were waxy in nature due to the semi-solid nature of the lipids [21]. However, the solid dispersions (SD-FP20K_(1:4)_ and SD-FP188_(1:4)_) prepared using PEG 20,000 and poloxamer 188 clearly showed solid particles which can be formulated into a tablet with by adding other excipients.

#### 3.4.2. Differential Scanning Calorimetry (DSC)

**Figure 3:**
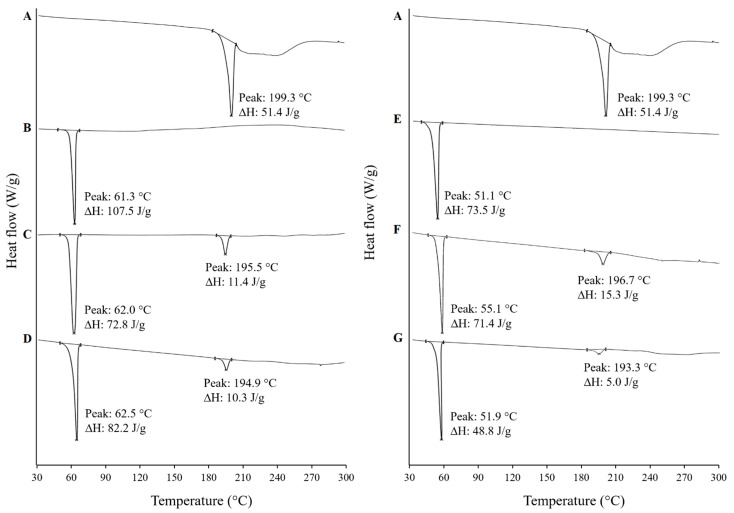
Differential scanning calorimetry (DSC) thermograms of (**A**) Supplied FFH, (**B**) Supplied PEG 20,000, (**C**) Physical mixture of FFH with PEG 20,000 at 1:4 ratio (PM-FP20K_(1:4)_), (**D**) Solid dispersion of FFH with PEG 20,000 at 1:4 ratio (SD-FP20K_(1:4)_), (**E**) Supplied poloxamer 188, (**F**) Physical mixture of FFH with poloxamer 188 at 1:4 ratio (PM-FP188_(1:4)_), (**G**) Solid dispersion of FFH with poloxamer 188 at 1:4 ratio (SD-FP188_(1:4)_).

Figure 3 shows the DSC thermograms of supplied FFH, PEG 20,000, poloxamer 188, the physical mixtures (PM-FP20K_(1:4)_ and PM-FP188_(1:4)_) and the solid dispersions (SD-FP20K_(1:4)_ and SD-FP188_(1:4)_). The DSC thermogram of supplied FFH showed a sharp endothermic melting peak at 199.3 °C with an enthalpy of fusion (ΔH) of 51.4 J/g indicating crystalline form I of FFH [41]. The DSC thermograms of supplied PEG 20,000 and poloxamer 188 showed an endothermic melting peak at 61.3 °C (ΔH-107.5 J/g) and 51.1 °C (ΔH-73.5 J/g), respectively, which are consistent with the literature [42,43]. The physical mixtures and solid dispersions produced using FFH and PEG 20,000/poloxamer at 1:4 weight ratio showed the endothermic peaks of the drug and respective carrier (PM-FP20K(1:4): FFH—195.5 °C, PEG 20,000—62.0 °C; PM-FP188_(1:4)_: FFH—196.7 °C, poloxamer 188- 55.1 °C; SD-FP20K_(1:4)_: FFH—194.9 °C, PEG 20,000—62.5 °C; and SD-FP188_(1:4)_: FFH—193.3 °C, poloxamer 188—51.9 °C) with a slight shift in peak position. The presence of melting endotherms of FFH and carriers, PEG 20,000 and poloxamer 188 in PM-FP20K_(1:4),_ PM-FP188_(1:4)_, SD-FP20K_(1:4)_, and SD-FP188_(1:4)_) indicates the crystalline nature of drug and carriers in the prepared physical mixtures and solid dispersions. A slight shift in the melting peak position represents the degree of drug excipient miscibility during melting [44].

#### 3.4.3. X-Ray Powder Diffraction (XRPD)

**Figure 4:**
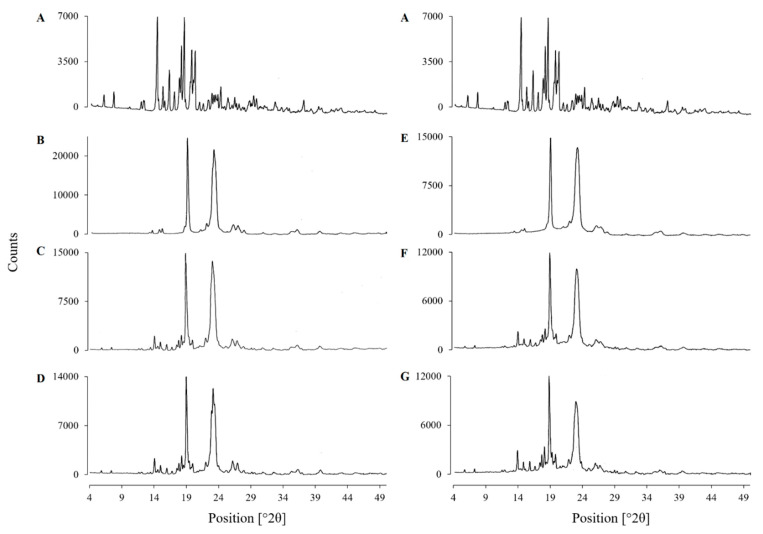
X-ray powder diffractograms (XRPD) of (**A**) Supplied FFH, (**B**) Supplied PEG 20,000, (**C**) Physical mixture of FFH with PEG 20,000 at 1:4 ratio (PM-FP20K_(1:4)_), (**D**) Solid dispersion of FFH with PEG 20,000 at 1:4 ratio (SD-FP20K_(1:4)_), (**E**) Supplied poloxamer 188, (**F**) Physical mixture of FFH with poloxamer 188 at 1:4 ratio (PM-FP188_(1:4)_), (**G**) Solid dispersion of FFH with poloxamer 188 at 1:4 ratio (SD-FP188_(1:4)_).

Figure 4 shows the XRPD patterns of supplied FFH, PEG 20,000, poloxamer 188, the physical mixtures (PM-FP20K_(1:4)_ and PM-FP188_(1:4)_) and the solid dispersions (SD-FP20K_(1:4)_ and SD-FP188_(1:4)_) with sharp diffraction peaks indicating their crystalline nature. The diffractogram of supplied FFH showed characteristic peaks [2θ] at 14.1°, 16.0°, 17.9°, 18.3°, 19.5° and 19.9°, matching with the diffraction pattern of crystalline FFH reported by Kumar et al. (2009) [41]. PEG 20,000 and poloxamer 188 showed intense diffraction peaks [2θ] at 19.1° and 23.2°, matching with the diffractograms reported by Windbergs et al. (2009) [43] and Ige, Baria, and Gattani (2013) [45], respectively. The physical mixtures (PM-FP20K_(1:4)_ and PM-FP188_(1:4)_) and solid dispersions (SD-FP20K_(1:4)_ and SD-FP188_(1:4)_) produced a diffraction pattern that exactly matched with the superimposed diffractograms of the supplied FFH and respective carrier (PEG 20,000/poloxamer 188) indicating the crystalline nature of drug in its solid dispersion/physical mixture form. The absence of new peaks in the prepared solid dispersions and physical mixtures ruled out the formation of new crystalline phase [46].

#### 3.4.4. Fourier Transform Infrared Spectroscopy (FTIR)

**Figure 5:**
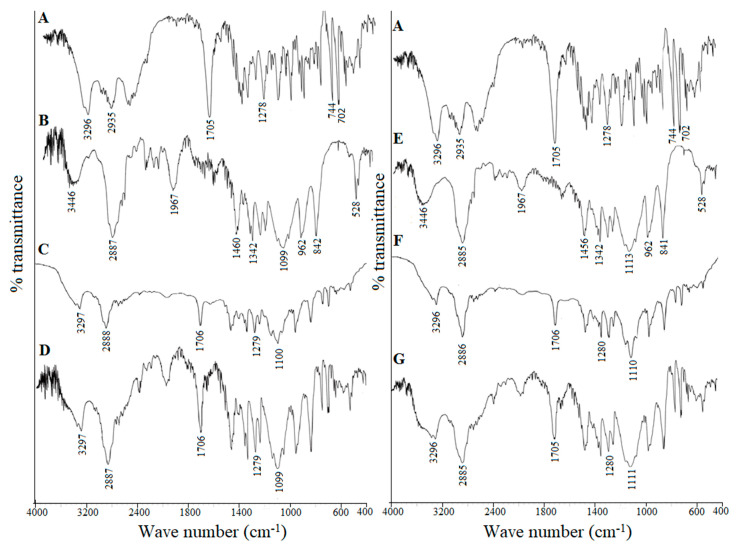
Fourier transform infrared (FTIR) spectra of (**A**) Supplied FFH, (**B**) Supplied PEG 20,000, (**C**) Physical mixture of FFH with PEG 20,000 at 1:4 ratio (PM-FP20K_(1:4)_), (**D**) Solid dispersion of FFH with PEG 20,000 at 1:4 ratio (SD-FP20K_(1:4)_), (**E**) Supplied poloxamer 188, (**F**) Physical mixture of FFH with poloxamer 188 at 1:4 ratio (PM-FP188_(1:4)_), (**G**) Solid dispersion of FFH with poloxamer 188 at 1:4 ratio (SD-FP188_(1:4)_).

Figure 5 shows the FTIR spectra of supplied FFH, PEG 20,000, poloxamer 188, the physical mixtures (PM-FP20K_(1:4)_ and PM-FP188_(1:4)_) and the solid dispersions (SD-FP20K_(1:4)_ and SD-FP188_(1:4)_). The FTIR spectrum of FFH showed characteristic peaks (cm^−1^) at 3296 (ν O–H alcohol/phenol), 2935 (ν O–H carboxylic acid), 1705 (ν C=O carboxylic acid), 1278 (ν C–N amine), 744 and 702 (δ C–H aromatic); PEG 20,000 at 3300 to 3600 (ν O–H), 2800 to 2900 (ν C–H of OC2H5) and 1000 to 1200 (ν C–O); poloxamer 188 at 3502 (ν O–H), 2885 (ν C–H), and 1112 (ν C–O). All the characteristic peaks of FFH and respective carrier (PEG 20,000/poloxamer) were conserved at the same position in their physical mixture (PM-FP20K_(1:4)_ and PM-FP188_(1:4)_) and the solid dispersions (SD-FP20K_(1:4)_ and SD-FP188_(1:4)_) indicating the absence of interactions between FFH and carrier in the physical mixtures and solid dispersions.

### 3.5. In Situ Intestinal Absorption Study

The oral bioavailability of FFH in rats was reported to be about 4.2% [47] due to its low intestinal permeability, and the involvement of intestinal P-gp [19,48,49]. The phase solubility and in vitro dissolution studies have revealed the solubility and dissolution rate enhancement of FFH with PEG 20,000 and poloxamer 188. To understand the influence of the PEG 20,000 and poloxamer 188 on fexofenadine permeation, in situ single pass intestinal perfusion study was conducted in rats and the results are shown in Table 3. The effective permeability coefficient of fexofenadine across rat intestine (P_eff(rat)_) from the dispersions of supplied FFH, solid dispersions SD-FP20K_(1:4)_ and SD-FP188_(1:4)_ was found to be 7.04 ± 0.56 (×10^−6^) cm/s, 14.40 ± 0.27 (×10^−6^) cm/s and 21.30 ± 0.45 (×10^−6^) cm/s, respectively.

The estimated human effective permeability coefficient (P_eff(human)_) and absorption rate constant (K_a_) values also indicate significantly (*p* < 0.05) higher rate of absorption of the drug from prepared solid dispersions compared to the supplied FFH. The enhancement ratio (ER) for SD-FP20K_(1:4)_ and SD-FP188_(1:4)_ was found to be 2.04 and 3.03, respectively which is greater than 1 indicating enhanced permeation of drug from the solid dispersions. Microscopic observation of the intestinal segments without and with treatment of supplied fexofenadine, SD-FP20K_(1:4)_ and SD-FP188_(1:4)_ (Figure S2, supplementary information) showed all layers of the intestine without any disruption to the epithelium indicating good biological acceptance of the carriers, PEG 20,000 and poloxamer 188 in the preparation of pharmaceutical formulations for oral administration.

Both SD-FP20K_(1:4)_ and SD-FP188_(1:4)_ were able to significantly enhance (*p* < 0.05) the intestinal permeability compared to that of supplied FFH dispersion. However, the extent of enhancement was significantly higher with non-ionic surfactant, poloxamer 188 than PEG 20,000 which could be attributed to the inhibition of P-gp efflux pump along with faster dissolution rate. Several studies have reported that poloxamer 188 inhibits P-gp mediated efflux activity and enhance the permeability of P-gp substrate drugs across rat intestinal segments [30,50]. Therefore, the present study suggests that poloxamer 188 could serve as a better carrier to improve the oral bioavailability of FFH by enhancing dissolution as well as intestinal permeability.

## 4. Conclusions

In this study, solid dispersions of fexofenadine hydrochloride were successfully prepared using PEG 20,000 and poloxamer 188 following fusion method. The phase solubility and in vitro dissolution studies revealed an improved solubility and dissolution rate of FFH with increased concentration of carriers compared to the supplied drug. The solid-state characterization using DSC and XRPD analysis suggested the crystalline nature of fexofenadine in the prepared solid dispersions. The FTIR observation supported the absence of the physicochemical interactions between drug carrier combinations studied. The in situ single pass intestinal perfusion study was carried out to evaluate the effect of the carriers in the intestinal absorption of fexofenadine from the prepared solid dispersions. Both the solid dispersions, SD-FP20K_(1:4)_ and SD-FP188_(1:4)_, showed 2–3 fold improvement in fexofenadine absorption compared to the control drug only dispersion. However, the extent of enhancement of intestinal absorption was significantly higher with poloxamer 188 than PEG 20,000 which could be attributed to the inhibition of P-gp efflux pump along with faster dissolution rate. The results of this study demonstrated that poloxamer 188 could serve as a better carrier to improve the oral bioavailability of FFH by enhancing dissolution as well as intestinal permeability.

## Figures and Tables

**Table 1:** Phase solubility and Gibbs-free energy of transfer (ΔG°_tr_) of fexofenadine hydrochloride in different concentrations of PEG 20,000 and poloxamer 188 water systems at 37 ± 0.5 °C (Mean ± SD, *n* = 3).

Concentration of Carrier (% *w/v*)	PEG 20,000		Poloxamer 188	
	Drug Solubility (mg/mL)	ΔG°_tr_ (J/mole)	Drug Solubility (mg/mL)	ΔG°_tr_ (J/mole)
0	1.45 ± 0.15	-	1.45 ± 0.15	-
5	3.14 ± 0.19	−1.99	4.06 ± 0.21	−2.66
10	5.06 ± 0.23	−3.22	5.73 ± 0.24	−3.55
15	7.16 ± 0.18	−4.12	6.90± 0.19	−4.03
20	7.99 ± 0.26	−4.40	9.32 ± 0.14	−4.80
25	10.02 ± 0.17	−4.99	10.65 ± 0.28	−5.14
30	11.78 ± 0.18	−5.40	12.27 ± 0.22	−5.51

**Table 2:** Summary of dissolution parameters for supplied fexofenadine hydrochloride, solid dispersions and physical mixtures prepared using PEG 20,000 and poloxamer 188.

Parameters	Q_15_	Q_120_	DE_15_	DE_120_	t_50%_
Supplied FFH	16.9 ± 1.2	38.9 ± 2.1	8.5 ± 0.6	24.3 ± 1.4	˃ 120
SD-FP20K_(1:1)_ *	55.1 ± 1.6	70.0 ± 1.9	27.5 ± 0.8	60.9 ± 1.8	˃ 120
SD-FP20K_(1:2)_ *	71.1 ± 2.2	78.3 ± 2.6	35.5 ± 1.1	70.7 ± 1.8	60
SD-FP20K_(1:4)_ *	80.6 ± 1.8	85.1 ± 1.5	40.3 ± 0.9	78.4 ± 1.8	15
PM-FP20K_(1:4)_ *	27.8 ± 2.8	56.7 ± 1.1	13.9 ± 1.4	40.3 ± 2.0	˃ 120
SD-FP188_(1:1)_ *	66.3 ± 1.8	78.2 ± 2.3	27.5 ± 0.9	68.9 ± 1.7	˃ 120
SD-FP188_(1:2)_ *	74.2 ± 1.9	84.7 ± 2.3	35.5 ±1.0	75.7 ± 1.9	60
SD-FP188_(1:4)_ *	80.7 ± 1.6	94.7 ± 1.9	40.3 ± 0.8	83.1 ± 1.6	15
PM-FP188_(1:4)_ *	31.1 ± 2.8	58.2 ± 1.8	13.9 ± 0.6	43.0 ± 1.4	˃ 120

Q_15_ and Q_120_—percent drug released at 15 and 60 min; DE_15_ and DE_120_: dissolution efficiency at 15 and 120 min; t_50%_—time (min) taken to release 50% of the drug, FFH, fexofenadine hydrochloride; SD-FP20K—solid dispersion of FFH with PEG 20,000, PM- FP20K—physical mixture of FFH with PEG 20,000, SD-FP188—solid dispersion of FFH with poloxamer 188, PM-FP188—physical mixture of FFH with poloxamer 188, respectively. * Numbers represent the weight/weight ratios of FFH/carriers.

**Table 3:** In situ absorption parameters of fexofenadine hydrochloride from the prepared solid dispersions across rat intestine (Mean ± SD; *n* = 3).

Formulation	P_eff(rat)_ × 10^−6^ (cm/s)	P_eff (human)_ × 10^−5^ (cm/s)	k_a_ (min^−1^)	ER
Supplied FFH	7.04 ± 0.56	2.61 ± 0.18	0.016 ± 0.001	-
SD-FP20K_(1:4)_	14.40 ± 0.27 ^†^	5.00 ± 0.08 ^†^	0.030 ± 0.001 ^†^	2.04 ^†^
SD-FP188_(1:4)_	21.30 ± 0.45 ^†^	7.26 ± 0.14 ^†^	0.044 ± 0.001 ^†^	3.03 ^†^

FFH, fexofenadine hydrochloride; SD-FP20K_(1:4)_—solid dispersion of FFH with PEG 20,000 at 1:4 weight ratio; SD-FP188_(1:4)_—solid dispersion of FFH with poloxamer 188 at 1:4 weight ratio; P_eff(rat)_—effective permeability coefficient in rat; P_eff(human)_—predicted effective permeability coefficient in human; K_a_—absorption rate constant; ER—enhancement ratio. ^†^ indicates significant difference at *p* < 0.05 against control.

## Supplementary Materials

The following are available online at https://www.mdpi.com/1999-4923/13/3/310/s1, Figure S1: Phase solubility diagrams for fexofenadine hydrochloride in the presence of (**A**) PEG 20,000 and (**B**) poloxamer 188 in water at 37 ± 0.5 °C (mean ± SD, *n* = 3), Figure S2: Histological sections of rat intestine: (**A**) without treatment, after single pass intestinal perfusion studies with (**B**) supplied fexofenadine hydrochloride, solid dispersions (**C**) SD-FP20K_(1:4)_ and (**D**) SD-FP188_(1:4)_ showing intact layers of intestine (Hematoxylin/Eosin stain, 100X- magnification).
