# Supplementary material for: Improved Dissolution Rate and Intestinal Absorption of Fexofenadine Hydrochloride by the Preparation of Solid Dispersions: In Vitro and In Situ Evaluation
Source: Pharmaceutics. 2021 Feb 27;13(3):310. doi: 10.3390/pharmaceutics13030310 (PMC7997449; doi:10.3390/pharmaceutics13030310)
Supplement: Supplementary file 1 [file pharmaceutics-13-00310-s001.pdf]

pharmaceutics

Supplementary Materials: Improved Dissolution Rate and Intestinal Absorption of Fexofenadine Hydrochloride by the Preparation of Solid Dispersions: In Vitro and In Situ Evaluation

Basanth Babu Eedara, Dinesh Nyavanandi, Sagar Narala, Prabhakar Reddy Veerareddy and Suresh Bandari

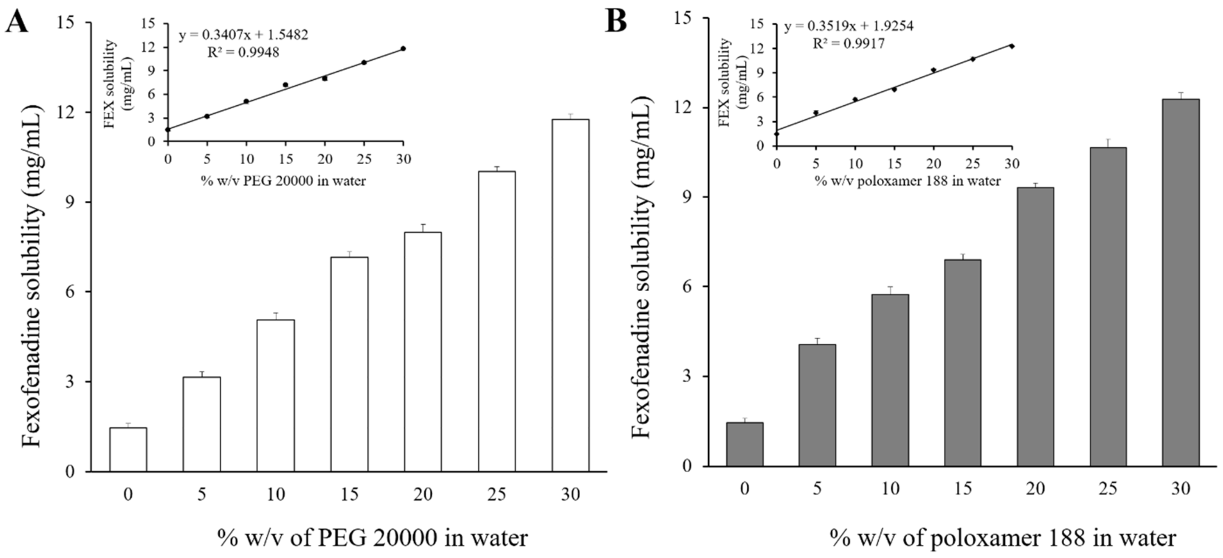

Figure S1. Phase solubility diagrams for fexofenadine in the presence of (A) PEG 20000 and (B) poloxamer 188 in water at 37 ± 0.5 °C (mean ± SD, $n = 3$).

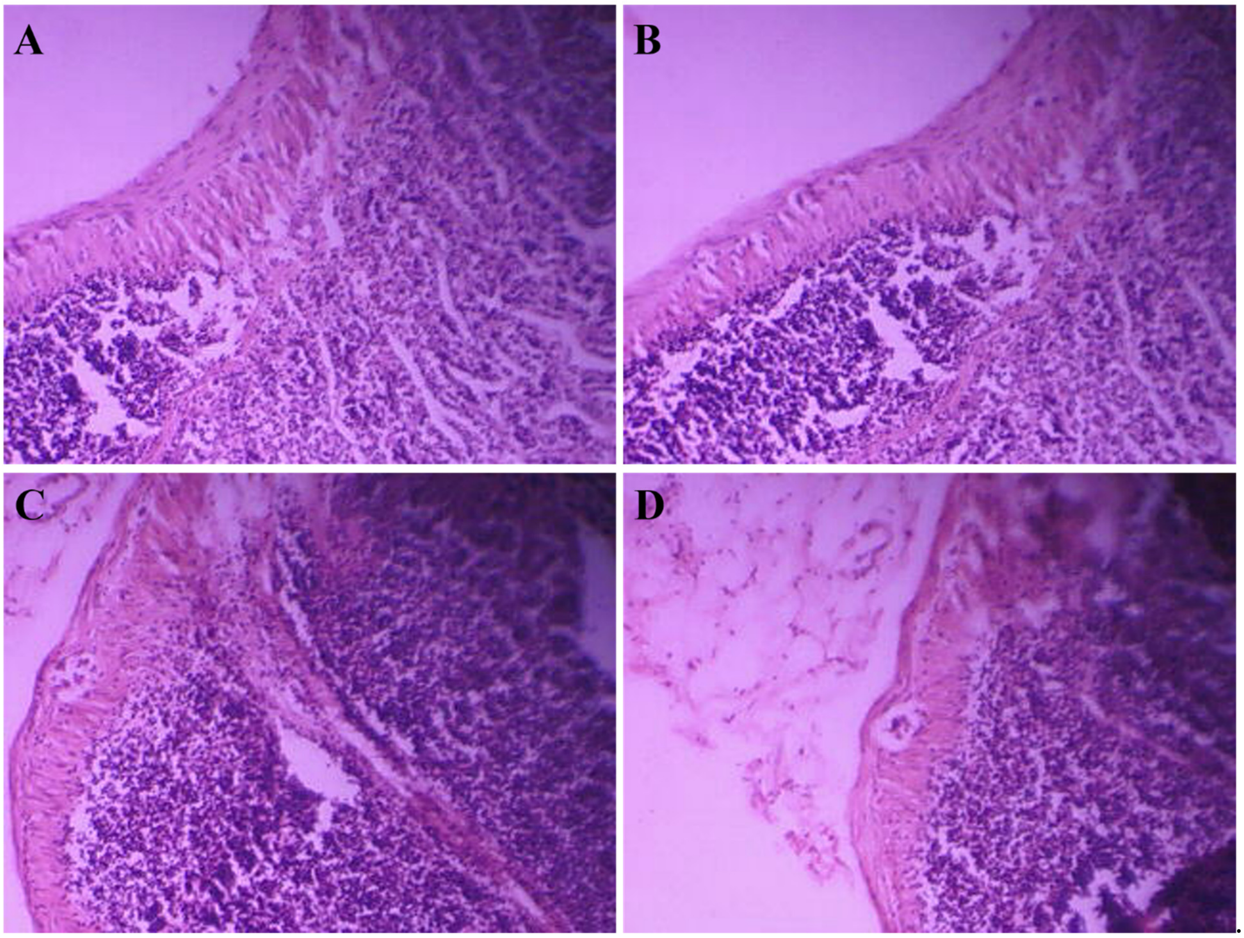

Figure S2. Histological sections of rat intestine: (A) without treatment, after single pass intestinal perfusion studies with (B) supplied fexofenadine, solid dispersions (C) SD-FP20K$_{(1:4)}$ and (D) SD-FP188$_{(1:4)}$ showing intact layers of intestine (Hematoxylin/Eosin stain, 100×- magnification).
